# Supplementary material for: Investigation of the reasons for delayed presentation in proliferative diabetic retinopathy patients
Source: PLoS One. 2024 Feb 29;19(2):e0291280. doi: 10.1371/journal.pone.0291280 (PMC10903851; doi:10.1371/journal.pone.0291280)
Supplement: S1 File — The Chinese original version and English translation of the ethics committee approval. (DOCX) [file pone.0291280.s001.docx]

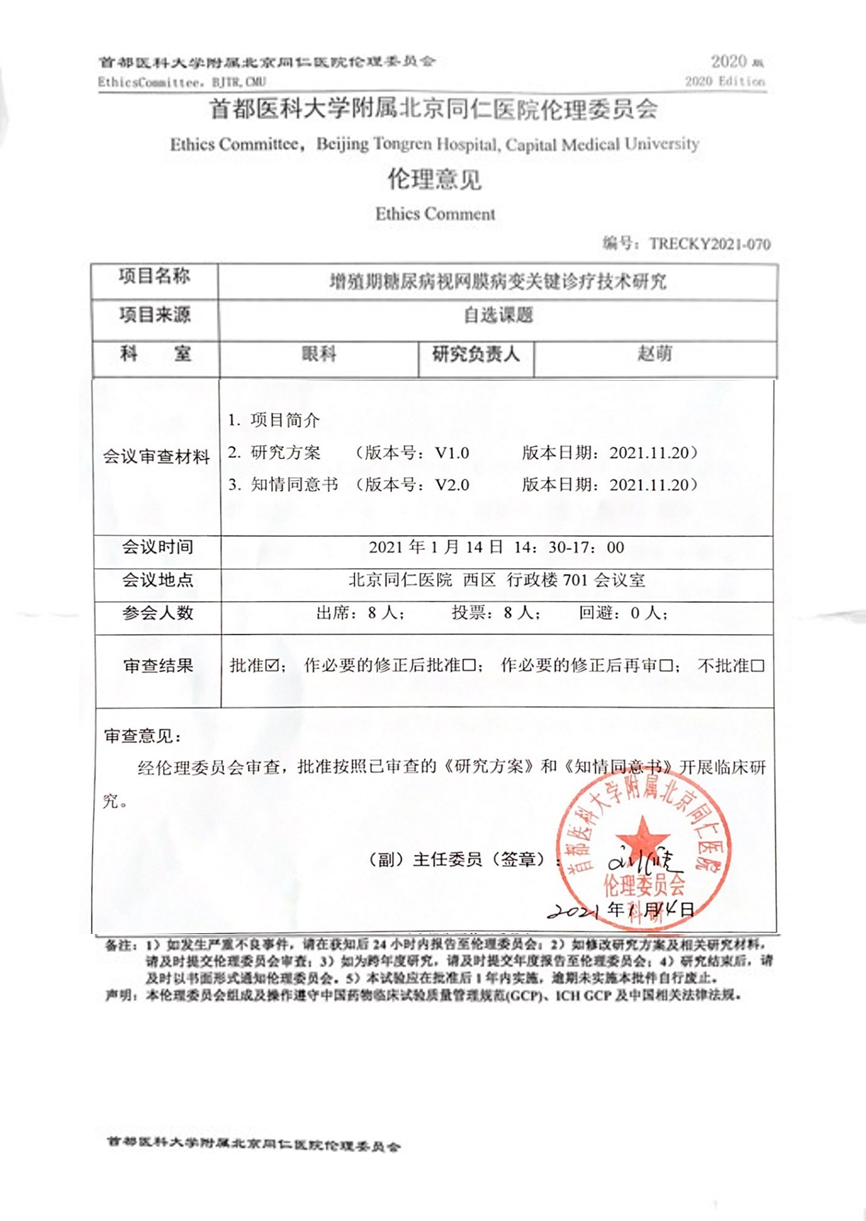


Translation version:

Title of the research: key issues in diagnosing and treating proliferative diabetic retinopathy

Fundings: no funding

The PI information: Meng Zhao ophthalmology department

The material provided to ethic committee: an introduction of the research. 2 protocol of the research (Version 1.0, 2021.11.20).3 informed consent (Version2, 2021.11.20)

Time of the meeting: January 14^th^, 2021, 14:30-17:00

The meeting was taken place at Xingzheng BUILDING of Tongren Hospital, room 701

Eight people participated and voted.

The ethics committee approved the study, please stick to the protocol and informed consent to carry out clinical research.

Approved date: January 14^th^ 2021

Signature of the chairman of the Beijing Tongren ethics committee: Jiagu Liu
