# Supplementary material for: Investigation of the reasons for delayed presentation in proliferative diabetic retinopathy patients
Source: PLoS One. 2024 Feb 29;19(2):e0291280. doi: 10.1371/journal.pone.0291280 (PMC10903851; doi:10.1371/journal.pone.0291280)
Supplement: S2 File — The informed consent of this study in Chinese. (DOCX) [file pone.0291280.s002.docx]

知情同意书

尊敬的病患：

您好！我们诚挚邀请您参与我们的研究项目，该项目旨在调查患有增生性糖尿病视网膜病变（PDR）的病患出现诊断延迟的原因。在您决定是否参与本研究之前，请您仔细阅读以下内容。本研究及此知情同意书已经获得北京同仁医院伦理委员会批准。

本知情同意书将向您介绍该研究的目的、步骤、获益、风险、不便或不适等，请仔细阅读后慎重做出是否参加研究的决定。当研究者向您说明和讨论知情同意书时，您可以随时提问并让他/她向您解释您不明白的地方。您可以与家人、朋友以及您的经治大夫讨论之后再做决定。您参与本研究是自愿的，可以选择是否参加。

本项研究的项目负责人是首都医科大学附属北京同仁医院的李继鹏医师。

1. 为什么要进行这项研究： 本研究旨在调查患有增生性糖尿病视网膜病变（PDR）的病患出现诊断延迟的原因。通过了解诊断延迟的原因，我们可以改进医疗护理，提高患者的治疗效果和生活质量。

2. 什么人可以参加研究：如果您满足以下条件：年龄在18至70岁之间，诊断为需要糖尿病视网膜病变治疗的增生性糖尿病视网膜病变患者，您可以参加本研究，。但如果您存在以下问题将无法参与本研究，包括：严重玻璃体出血，无法判断是否为PDR引起；双眼都因PDR接受了玻璃体切割手术；目前不需要激光或玻璃体切割手术，仅需要密切随访观察；无法填写调查问卷；患有青光眼、视神经疾病或黄斑脱离等可能引起不可逆视力损害的病患；在北京COVID-19封锁期间被限制出行，因DR患者的预约可能受限而导致治疗延迟。

3. 多少人会参加这项研究： 我们计划纳入150名患有增生性糖尿病视网膜病变的病患作为研究对象。

4. 研究是怎样进行的：

研究包括填写调查问卷，接受眼部检查并记录病史和医疗情况。我们会根据您的情况进行问卷调查，以了解您对糖尿病视网膜病变的认知、治疗态度、难处等方面的情况。眼部检查将帮助我们确定糖尿病视网膜病变的严重程度和下一步治疗。

感谢： 在此，我们要特别感谢所有参与本研究的病患，他们的贡献对研究的成功至关重要。同时，我们还要感谢北京同仁医院在数据收集过程中的支持。

5. 如果参加这项研究，您的责任： 如果您决定参加本研究，您需要填写调查问卷，并配合进行眼部检查。您的信息将被严格保密，仅用于研究目的。

6. 参加这项研究对受试者日常生活的影响： 您需要花费一些时间填写调查问卷，并前往医院进行眼部检查。我们将尽量安排您的时间，以减少对您日常生活的影响。

7. 如果参加有什么样的风险： 参与本研究可能涉及填写个人隐私信息的调查问卷。我们将采取措施保护您的个人隐私和信息安全。

8. 如果不参加会影响治疗吗： 不参加本研究不会影响您在医疗机构的治疗和待遇。您的选择不会对您的医疗关系产生任何影响。

9. 参加可能的受益： 通过参与本研究，您有机会为改善增生性糖尿病视网膜病变患者的治疗情况和医疗护理质量做出贡献。您的参与将有助于我们更好地了解诊断延迟的原因，从而提高患者的生活质量。

10. 保密： 您的个人信息将被严格保密，仅用于研究目的，不会用于商业用途。

11. 是否一定要参加完成这一研究： 您的参与是自愿的，您可以随时决定退出研究而不受任何影响。

12. 如果有疑问或出现问题联系谁： 如果您对本研究有任何疑虑、问题或需要进一步了解，请随时与我们联系。我们会为您提供详细的信息和帮助。联系方式：李医生58268254

感谢您的时间和合作！

研究团队：北京同仁眼科中心 眼底科

【病患签名】 【日期】

如果患者不能完成签名，代理人签字：

【研究人员签名】 【日期】
