# Supplementary material for: Investigation of the reasons for delayed presentation in proliferative diabetic retinopathy patients
Source: PLoS One. 2024 Feb 29;19(2):e0291280. doi: 10.1371/journal.pone.0291280 (PMC10903851; doi:10.1371/journal.pone.0291280)
Supplement: S3 File — The English translation of the informed consent of this study. (DOCX) [file pone.0291280.s003.docx]

Informed Consent Form

Version 2 2021.11.20

Dear Participant,

Before deciding whether to participate in this research, please carefully read the following information. If you have any questions or need further clarification about any of the following points, please feel free to contact us.

1. Purpose of the Study: This study aims to investigate the reasons for delayed presentation in patients with proliferative diabetic retinopathy (PDR). By understanding the factors contributing to delayed diagnosis and treatment, we aim to enhance medical care and improve patient outcomes.

2. Eligibility for Participation: You are eligible to participate if you meet the following criteria: aged between 18 and 70 years, diagnosed with proliferative diabetic retinopathy (PDR) requiring treatment. Ineligibility criteria include: severe vitreous hemorrhage of unclear cause, bilateral vitrectomy due to PDR, absence of immediate treatment need, inability to complete the questionnaire, presence of conditions causing irreversible vision loss, and lockdown during the COVID-19 pandemic affecting appointment availability.

3. Number of Participants: We plan to include a total of 150 patients diagnosed with proliferative diabetic retinopathy (PDR) who have experienced delayed diagnosis.

4. Conduct of the Study: The study involves completing a questionnaire and undergoing an eye examination. The questionnaire will help us understand your knowledge of diabetic retinopathy, your attitude towards treatment, and any challenges you face. The eye examination will help determine the severity of your condition and treatment needs.

5. Your Responsibilities if You Decide to Participate: If you choose to participate in this research, you will be required to complete a questionnaire and cooperate in undergoing an eye examination. Your information will be kept strictly confidential and will only be used for research purposes.

6. Impact on Participant's Daily Life: Participating in this research will require you to spend some time filling out a questionnaire and visiting the hospital for an eye examination. We will try to accommodate your schedule to minimize the impact on your daily life.

7. Potential Risks of Participation: Participating in this research may involve filling out a questionnaire that contains personal privacy information. We will take measures to protect your personal privacy and the security of your information.

8. Impact of Non-Participation on Treatment: Not participating in this research will not affect your treatment and care at the medical institution. Your decision will not have any impact on your medical relationship.

9. Potential Benefits of Participation: By taking part in this research, you have the opportunity to contribute to improving the treatment and medical care quality for patients with proliferative diabetic retinopathy (PDR). Your participation will help us better understand the reasons for diagnostic delays, thus enhancing patients' quality of life.

10. Confidentiality: Your personal information will be strictly confidential and used solely for research purposes, without any commercial use.

11. Voluntary Participation: Your participation in this research is voluntary, and you can decide to withdraw from the study at any time without any consequences.

12. Contact for Questions or Concerns: If you have any concerns, questions, or need further information about this research, please don't hesitate to contact us. We will provide you with detailed information and assistance.

Thank you for your time and cooperation!

Research Team

Participant's Signature: ________________________ Date: ________________________

Researchers’ Signature: ________________________ Date: ________________________
