# Supplementary material for: Investigation of the reasons for delayed presentation in proliferative diabetic retinopathy patients
Source: PLoS One. 2024 Feb 29;19(2):e0291280. doi: 10.1371/journal.pone.0291280 (PMC10903851; doi:10.1371/journal.pone.0291280)
Supplement: S4 File — The English translation of the questionnaire of the study. (DOCX) [file pone.0291280.s004.docx]

Appendix

The questionnaire for PDR patients with delayed presentation

| Basic information | | | | | |
| --- | --- | --- | --- | --- | --- |
| 1 | name | |  | | |
| 2 | gender | |  | | |
| 3 | Date of birth | |  | | |
| 4 | Date of presentation | |  | | |
| Question about past history | | | | | |
| 5 | Duration of DM (m) | |  | | |
| 5.1 | If duration of DM is less than 6 months:were you diagnosed with DM after presentation at ophthalmic clinics for VA deterioration?  1 yes 2 no | |  | | |
| 5.2 | If the duration of DM is greater or equal to 6 months: do you visit your internal medicine physicians regularly ?  1 yes 2 no | |  | | |
| 6 | Known DM systemic complications | | | | 1 present, 2 absent, 3 not know |
| 7 | cerebral vascular disease | | | |  |
| 8 | Chronic kidney disease | | | |  |
| 9 | Coronary disease | | | |  |
| 10 | Diabetic foot | | | |  |
| 11 | other | | | |  |
| 12 | Have you ever rejected suggestion for DR screening?  1 yes 2 no | | | |  |
| 13 | Have you received cataract surgery?  1 yes 2 no | | | |  |
| 13.1 | If 13 answer is “yes”: did you have cataract surgery before the onset of blurring?  1 yes 2 no | | | |  |
| 13.1.1 | If 13.1 answer is “yes”: did you have a period of “good vision” before the onset of blurring?  1 yes 2 no | | | |  |
| 14 | Have you received laser treatment for diabetic retinopathy?  1 yes 2 no | | | |  |
| 14.1 | If 14 answer is “yes”: which of the following statement is your condition?  1 I had laser therapy before the onset of ocular symptom of DR.  2 I had laser therapy after the onset of DR ocular symptom and was told the laser therapy was completed.  3 I had laser therapy after the onset of DR ocular symptom and was not told the laser therapy was completed.  4 I had laser therapy after the onset of DR ocular symptom and was told the laser therapy was not completed. | | | |  |
| 15 | Have you received intravitreal injection of medication for diabetic retinopathy?  1 yes 2 no | | | |  |
| Section 1 knowledge on diabetic retinopathy: | | | | | |
| 16 | Do you know that DR can lead to blindness if treated too late?  1 yes 2 no | | | |  |
| 17 | Do you know that diabetic patients need to visit ophthalmologist regularly to detect DR?  1 yes 2 no | | | |  |
| 18 | Do you know that DR can be found in patients with excellent vision?  1 yes 2 no | | | |  |
| 19 | Multi-choice question:  Where did you learn the above information about DR?  A media B Internal medicine physicians C ophthalmologists D relatives or friends | | | |  |
| Section 2 altitude towards DR screening or treatment: | | | | | |
| 20 | Multi-choice question:  Why did you refuse to take DR screening or treatment?  1 I did not know that I had DM.  2 My visual was excellent and I did not need fundus examination.  3 I was afraid that the doctor could find something going wrong on my retina,  4 The examination was too painful and discomfort.  5 I was occupied with work/ taking care of family members.  6 I can’t go to hospital by myself.  7 There was no DR screening available in my neighborhood,  8 I was hospitalized for other systemic abnormalities or diabetic systemic complications.  9 I was told that taking Chinese traditional medicine can cure DM. | | | |  |
| 21 | Have you refused the suggestion for regular DR screening or DR treatment?  1 yes 2 no | | | |  |
| 22 | Did you ever been informed that you have diabetic retinopathy?  1 yes 2 no | | | |  |
| 23 | Have you checked your eyes for diabetic retinopathy yearly?  1 yes 2 no | | | |  |
| 23.1 | If 23 answer is “yes”: have you checked your eyes for diabetic retinopathy within the latest six months?  1 yes 2 no | | | |  |
| 24 | Do you have insurance coverage for DR screening or DR treatment?  1 yes 2 no | | | |  |
| 25 | Have you visit your physician regularly (1-2month)?  1 yes, I have my systemic complication and control of glucose evaluation on regular schedule.  2 yes, I visit my doctor and only get the prescription without further examination.  3 no, I stick to traditional Chinese medicine clinic and do not visit the physician.  4 no. I get my medication from other (relatives) and don not visit the physician. | | | |  |
| 26 | Do you have yearly complete checkup?  1 yes 2 no | | | |  |
| 26.1 | If 26 answer is no: when did you have the latest complete checkup? | | | |  |
| 27 | Did you have severe diseases other than DM in the recent two years? (eg tumor, infection?)  1 yes 2 no | | | |  |
| Section 3 : investigationon difficulties in real-life that impact their willingness to seek DR diagnosis and treatment. | | | | | |
| 28 | | Multi-choice question:  What do you think impact your willingness to stick to DR screening or treatment?  1 transportation inconvenience  2 I can’t afford the examination or treatment.  3 I can’t go to hospital and check my eyes by myself.  4 I am occupied by work or family issue.  5 I am hospitalized for systemic abnormalities.  6 I am afraid of finding DR .  7 I think the DR treatment is completed. | |  | |
| Results from outpatient clinic examinations | | | | | |
| HbA1c | | | |  | |
| BUN | | | |  | |
| creatinine | | | |  | |
| Urine protein | | | |  | |
| Presence of the following condition in consultation with physician | | | | | |
| Use of insulin | | | |  | |
| HTN | | | |  | |
| Cardiovascular disease | | | |  | |
| Cerebral vascular disease | | | |  | |
| Chronic kidney disease | | | |  | |
| Diabetic foot | | | |  | |
| Ocular findings | | | | | |
| Duration of ocular symptom? | | | |  | |
| Severity of DR on the contralateral eyes  1 required only PRP  2 required PPV  3 blindness | | | |  | |
| Severity of DR on the investigated eyes  1 required only PRP  2 required PPV  3 blindness | | | |  | |
| Presence of NVG or NVI on the investigated eyes  1 yes 2 no | | | |  | |
| Evaluation of PRP  1 no  2 not complete  3 complete | | | |  | |
