# Supplementary material for: Investigation of the reasons for delayed presentation in proliferative diabetic retinopathy patients
Source: PLoS One. 2024 Feb 29;19(2):e0291280. doi: 10.1371/journal.pone.0291280 (PMC10903851; doi:10.1371/journal.pone.0291280)
Supplement: Attachment — Submitted filename: response to reviewer second.docx [file pone.0291280.s006.docx]

**Dear Reviewers,**

We thank you for your thoughtful and constructive comments on our manuscript entitled " Investigation of the reasons for delayed presentation in proliferative diabetic retinopathy patients."

We have carefully considered your feedback and have made the following revisions to the manuscript.

In response to Reviewer #1's comments, we have added the following information to the **introduction:**

- Inflammation is a common feature of type 2 diabetes and its complications, including diabetic retinopathy. (line 77)
- Specifically, inflammation has been shown to be associated with the progression of diabetic neuropathy, diabetic retinopathy, and diabetic nephropathy. (line 78)
- Early detection of diabetes and its complications, including diabetic retinopathy, could improve effective management of the disease and its complications. (line 80)

We have also added the following references to support these claims:

1. Swiss Med Wkly. 2019;149:w20139. doi: 10.4414/smw.2019.20139 (line 79)
2. Eur J Clin Invest. 2020;50(3):e13206. doi: 10.1111/eci.13206 (line 80)
3. Preprints 2023, 2023060202. doi:10.20944/preprints2023060202.v1 (line 79)
4. Front Immunol. 2020;11:583687. doi: 10.3389/fimmu.2020.583687. eCollection 2020 (line 80)
5. Postgrad Med. 2023;135(5):519-523. doi: 10.1080/00325481.2023.2214058 (line 80)

We have also revised the **discussion** section to address Reviewer #1's comments. Specifically, we have added the following information:

- We have emphasized the potential role of inflammation in accelerating the progression of diabetic retinopathy in patients with poorly controlled diabetes. (line 482-485,506,554)
- We have discussed the findings of previous studies that have shown a relationship between inflammation and the progression of diabetic retinopathy. (lines 482-485)
- We have suggested that future studies should investigate the potential use of inflammation markers to identify patients at risk for progression of diabetic retinopathy. (lines 803-806)
- In the methods and discussion limitations section, we have added the following sentence:

In line 163 and 806-807, we have added the following sentence to clarify that we did not include gestational diabetes mellitus patients in our study and therefore could not study the relationship between atrial conduction time and P-wave dispersion and DR progression.

We have not received any comments from Reviewer #3.

In addition to the specific revisions mentioned above, we have also made the following general revisions to the manuscript:

- We have clarified the language and structure of the manuscript to improve readability.
- We have corrected any errors in grammar or spelling.
- We have added additional information to support the claims made in the manuscript.

We believe that these general revisions have improved the overall quality of the manuscript.

We believe that these revisions have addressed the concerns raised by you. We hope that you will find the revised manuscript to be satisfactory.

Thank you for your time and consideration.

Sincerely,

Jipeng Li

Beijing Tongren Eye Center
